# DNA Sequence Heterogeneity of *Campylobacter jejuni* CJIE4 Prophages and Expression of Prophage Genes

**DOI:** 10.1371/journal.pone.0095349

**Published:** 2014-04-22

**Authors:** Clifford G. Clark, Patrick M. Chong, Stuart J. McCorrister, Philip Mabon, Matthew Walker, Garrett R. Westmacott

**Affiliations:** 1 Enterics Diseases, Bacteriology and Enterics, National Microbiology Laboratory, Public Health Agency of Canada, Winnipeg, Canada; 2 Mass Spectrometry and Proteomics Core Facility, National Microbiology Laboratory, Public Health Agency of Canada, Winnipeg, Canada; 3 Bioinformatics Core Facility, National Microbiology Laboratory, Public Health Agency of Canada, Winnipeg, Canada; Iowa State University, United States of America

## Abstract

*Campylobacter jejuni* carry temperate bacteriophages that can affect the biology or virulence of the host bacterium. Known effects include genomic rearrangements and resistance to DNA transformation. *C. jejuni* prophage CJIE1 shows sequence variability and variability in the content of morons. Homologs of the CJIE1 prophage enhance both adherence and invasion to cells in culture and increase the expression of a specific subset of bacterial genes. Other *C. jejuni* temperate phages have so far not been well characterized. In this study we describe investigations into the DNA sequence variability and protein expression in a second prophage, CJIE4. CJIE4 sequences were obtained de novo from DNA sequencing of five *C. jejuni* isolates, as well as from whole genome sequences submitted to GenBank by other research groups. These CJIE4 DNA sequences were heterogenous, with several different insertions/deletions (indels) in different parts of the prophage genome. Two variants of a 3–4 kb region inserted within CJIE4 had different gene content that distinguished two major conserved CJIE4 prophage families. Additional indels were detected throughout the prophage. Detection of proteins in the five isolates characterized in our laboratory in isobaric Tags for Relative and Absolute Quantitation (iTRAQ) experiments indicated that prophage proteins within each of the two large indel variants were expressed during growth of the bacteria on Mueller Hinton agar plates. These proteins included the extracellular DNase associated with resistance to DNA transformation and prophage repressor proteins. Other proteins associated with known or suspected roles in prophage biology were also expressed from CJIE4, including capsid protein, the phage integrase, and MazF, a type II toxin-antitoxin system protein. Together with the results previously obtained for the CJIE1 prophage these results demonstrate that sequence variability and expression of moron genes are both general properties of temperate bacteriophages in *C. jejuni*.

## Introduction

Prophages were first demonstrated in *Campylobacter* spp. in the 1960s and 1970s, but it was only in 2005 that whole genome sequencing confirmed their presence in *C. jejuni, C. coli*, and *C. lari* [1]. These prophages appear to be structurally diverse and mobile within the genome [2]; a consequence that has been demonstrated to arise from this is changes in PFGE patterns [3]. They can be induced from the bacterial genome by lytic phage predation and thus cause genome rearrangements [4] and they carry genes for extracellular DNases responsible for a dramatic reduction in the frequency of natural transformation [5], [6]. We have previously found sequence variability in *Campylobacter jejuni* integrated element 1 (CJIE1) prophages [7], [8] that was associated with differences in virulence among isolates. CJIE1 prophages were associated with increased adherence and invasion to INT-407 cells in culture of the prophage-carrying isolates [9], phenotypes associated with differential expression of a subset of bacterial genes and proteins [10]. Like prophages in *Escherichia coli* and *Salmonella enterica* [11], it is possible that *C. jejuni* prophages may carry effectors or other proteins that are virulence factors expressed by the host bacterium. If prophages of *C. jejuni* were found to play roles similar to those of *E. coli* and *Salmonella*, an understanding of the contribution of these prophages to virulence and host adaptation could be critical for surveillance, source tracking, and control of the subset(s) of *C. jejuni* responsible for human disease. The investigations that form the basis of this report were designed to query whether there was sequence variability in a second *C. jejuni* prophage, CJIE4, and to investigate whether variably carried genes were expressed as proteins and could therefore affect the biology of the host bacterium.

CJIE4 was observed by Fouts et al. to exhibit minimal DNA sequence similarity to CJIE1 from isolate RM1221 and greater similarity to both CJIE2 from *C. jejuni* RM1221 and CLIE1 from *C. lari* RM2100 [1]. CJIE4 prophages were found in 21% of the *C. jejuni* tested and showed rather limited diversity in gene content by comparative genomic analysis using DNA microarrays [2]. Whole genome sequencing of *C. jejuni* strain 414 also demonstrated the presence of a prophage homologous to CJIE4, though less than 75% of the RM1221 CJIE4 genes were present [12]. Similar variability in CJIE4 content was reported by Pittenger et al. [13] in comparative genomic hybridization experiments. A prophage homologous to CJIE4 was also found in *C. jejuni* S3 [14].

Unfortunately, our laboratory has been so far unsuccessful at inducing and propagating *C. jejuni* prophages, consistent with the findings of at least one other group [15]. As an alternative strategy to investigate the role(s) and importance of these phages we have characterized prophage sequence variation by sequencing the prophage genomes from five very different clinical isolates of *C. jejuni* and comparing the results to CJIE4 sequences available in sequence databases. CJIE4 prophage sequences were found to be variable, containing a large indel with differential gene content in the middle of the genome and several other differentially carried genes throughout. Proteins encoded by genes within both major variants of the large central indel were detected, as were additional proteins encoded by conserved prophage genes. However, an examination of the putative functions of these proteins based on homology to known proteins suggested that most or all of the moron proteins carried by CJIE4 prophages may be associated with the function or biology of the phages themselves rather than having an influence on the biology of the host bacterium.

## Materials and Methods

### Isolates and growth conditions

The five isolates used for the study were associated with the investigation into the spring 2000 *Campylobacter* and *E. coli* outbreak in Walkerton, Ontario, Canada [16], [17]. All isolates previously tested positive in PCR assays for the presence of three genes from CJIE4 [8]. One of the goals of the study was to assess diversity in CJIE4 prophages; therefore, isolates with different serotypes and isolated in different years were chosen where possible [16]. The Oxford multi-locus sequence types (STs) for the five isolates are: 00-2425, ST 21; 00-0949 and 01-1512, ST 8; 00-6200, ST 806; 00-1597, ST 930.

*C. jejuni* isolates were kept for long-term storage in either 20% skim milk or glycerol peptone water (25% v/v glycerol, 10 g/L neopeptone, 5 g/L NaCl) at −80°C. For use, *C. jejuni* isolates with a low passage number were retrieved from storage at −80°C, plated to Oxoid Mueller-Hinton agar (Oxoid Inc.) containing 10% sheep red blood cells, and grown for 48–72 h at 37°C under a microaerobic atmosphere (5% O_2_, 10% CO_2_, 85% N_2_).

### DNA Sequencing and annotation

Prophages were sequenced by PCR amplification and DNA sequencing of 2–3 kb fragments as outlined previously [7], [8]. Sequences were compiled using the SeqMan program in the DNAStar Lasergene 8 package. Open reading frames were initially detected using Edit Seq in the DNAStar Lasergene 8 package, and translated products were annotated using the following software: Pfam: http://pfam.sanger.ac.uk/search?tab=searchSequenceBlock; HHPred: http://toolkit.tuebingen.mpg.de/hhpred; BLAST with default settings (NCBI home page http://www.ncbi.nlm.nih.gov; PSI-BLAST with the Entrez query “viruses [orgn]” (NCBI). Annotations were checked and corrected using GenDb and the annotated prophage genomes were used to create GenBank submissions.

### Accession numbers for DNA sequences submitted to GenBank

The whole genome sequence of isolate 00-2425 is CP006729. The accession numbers for the CJIE4 prophages from the other four isolates are as follows: 00-0949, KF751793; 00-1597, KF751794; 00-2425, KF751795; 00-6200, KF751796; 01-1512, KF751797.

### Sequences obtained from GenBank

Full-length sequences of the CJIE4 homologs from isolates 00-0949 and 00-2425 were used to perform a BLASTn search of the nr database in Genbank. The latest date that sequences were checked was June 3, 2013.

Accession numbers for contiguous full length sequences in *C. jejuni* were: **RM1221**, NC_003912.7; **S3**, NC_017281.1; **140-16**, AIPF0100006.1; **1997-4**, AIOW01000013.1; **414**, ADGM01000016.1; **84-25**, AANT02000001.1. In other cases more than one contig was required to obtain coverage of the homologous prophage; in some cases, contigs containing only one or a few loci were assumed to be part of the larger prophage based on synteny with one or more of the five prophages sequenced for this study. Included were *C. jejuni* strains: **DFVF1099**, ADHK01000002.1, ADHK01000040.1; **1997-14**, AIPA01000003.1, AIPA01000079.1, AIPA01000094.1, AIPA01000120.1, AIPA01000183.1; **51037**, NZ_AIPB1000015.1, NZ_AIPB1000046.1, NZ_AIPB1000089.1; **51494**, AINZ01000009.1, AINZ01000039.1, AINZ01000066.1, AINZ01000122.1; **87459**, AIPE01000015.1, AIPE01000029.1, AIPE01000086.1. A single *C. coli* sequence from strain **2548** (AIML01000004.1, AIML010000028.1, AIML010000068.1, AIML010000098.1) was used.

### PCR for verifying the location of the CJIE4 prophage within isolate 01-1512

All PCR reactions were done using DNA extracted from bacteria using a Gentra Systems PUREGENE DNA Isolation kit (Qiagen) according to the instructions of the manufacturer. PCR reactions were run using reagents from FastStart Taq DNA Polymerase kits (Roche). For each reaction the following parameters were used: final MgCl_2_ concentrations of 2.0 mM, 0.2 mM of each dNTP, 0.5 µM of each primer, and 2.5 U of FastStart DNA polymerase. PCR reactions were performed using a Perkin Elmer 9700 thermocycler.

PCR primers were developed on the basis of the location of the CJIE4 prophage within the draft whole genome sequence of *C. jejuni* isolate 01-1512, in which the prophage was inserted between genes encoding homologs of CJE0341c (upstream) and Cj0339c (downstream). Within each primer pair, one primer was located within a CJIE4 gene and one was located in the adjacent genomic DNA in order to unambiguously locate the genomic insertion site. Upstream primers were CJIE4uF 5′ GAG ATC TTT TTG CCT TGG GAC TT 3′ located in the homolog of CJ0341c (NCTC11168; CJE0386 in RM1221) and CJIE4uR 5′ TCG ATG ACA TGT GAA CGC TTG AT 3′ located within the homolog of CJE1418 (RM1221 CJIE4). These primers had an optimal annealing temperature of 50.8°C and produced a 907 bp product. Cycle conditions for the primer pair were: initial denaturation at 94°C for 5 min; 35 cycles of 94°C for 30 s, 50.8°C for 1 min, 72°C for 1 min; final extension at 72°C for 7 min; 4°C until reactions were analyzed.

Primers made to the other end of the CJIE4 prophage were CJIE4dF 5′ TCG GTG TAT GGG CTG AAT 3′ located in the intergenic region between homologs of CJE1472 and 1473 (RM1221 homologs) and CJIE4dR 5′ TAA AAT GGG TGA GTT GGT GAG TAA 3′ located within Cj0339 (NCTC11168; CJE0384 in RM1221). This primer pair had an optimal annealing temperature of 48.9°C and produced a 1544 bp amplicon. Cycle conditions for the primer pair were: initial denaturation at 94°C for 5 min; 35 cycles of 94°C for 30 s, 48.9°C for 1 min, 72°C for 2 min; final extension at 72°C for 7 min; 4°C until reactions were analyzed. Reaction products were analyzed by submarine electrophoresis using 1.6% agarose gels and stained with GelRed Nucleic Acid Stain (Cedarlane).

### Preparation of iTRAQ-labeled proteins for comparative proteomics analysis

Total cellular proteins were prepared, modified, and labelled with iTRAQ reagents as previously described [10]. Bacteria were recovered after 48 h growth on Mueller-Hinton agar plates with sterile Dulbecco's PBS, pH 7.0–7.2 (Gibco, Invitrogen), washed once with PBS, and suspended in sterile high-quality 18 MΩ (MilliQ) water. Acid-washed 212–300 µm glass beads (Sigma-Aldrich Canada Ltd.) were added to the suspension, vortexed, and then boiled for 5 min. Proteins were released from the boiled cells by several rounds of vortexing followed by shaking gently for 5 min on a vortexer fitted with a 12 place Ambion Vortexer Adapter for Genie 2 Vortex Mixer attachment (Applied Biosystems Canada). After centrifugation for 1 min at 3000 rpm (664 × g) the protein-containing supernatant was collected into a sterile 15 ml centrifuge tube. Protein preparations were used immediately or stored at −80°C for up to two months. The protein concentration of each preparation was estimated using a Pierce Protein Assay kit (Fisher Scientific) according to the manufacturer's protocol.

Protein modification and digestion in cartridges was done according to previously published methods [10], [18]. Crude protein suspensions containing 100 µg of protein were dried in a Savant DNA120 SpeedVac Concentrator (Fisher Scientific). Proteins were solubilized in 50 µl of freshly made SDS solubilization buffer (4% SDS, 50 mM HEPES buffer pH 8.3, 100 mM DTT) by heating at 95°C for 5 min. Urea Exchange Buffer (UEB; 0038 M urea in 50 mM HEPES, pH 8.3) was added to dilute the protein suspensions, proteins were added to Nanosep 10 K cartridges (VWR International LLC) and the buffer was exchanged with fresh UEB by centrifugation of the protein mix and dilution with fresh UEB. Proteins were then alkylated by adding 100 µl of 50 mM iodoacetamide (IAA Reagent, Sigma-Aldrich) in UEB, shaking for 5 min at RT, and incubation for 20 min without shaking in the dark. After removal of iodoacetamide by exchanging buffer three times with UEB alone, the UEB buffer was exchanged twice with 150 µl of 50 mM HEPES, pH 8.3. DNA was removed by the incubation with 50 µl of Benzonase (Sigma-Aldrich) solution (20 U/µl Benzonase in 42 mM HEPES, pH 8.3 containing 2 mM MgCl_2_), shaking at 600 rpm for 2 min at RT and incubation without shaking for 30 min at RT. After this incubation, the cartridge was washed three times with 100 µl of 50 mM HEPES, pH 8.3.

Proteins were digested ON with 5 µl (5 µg) trypsin (Trypsin Gold, mass spectrometry grade, Promega) in the Nanosep 10 cartridges. Following digestion the tryptic peptides were recovered from the cartridge, dried, and suspended in 30 µl 100 mM HEPES, pH 8.3. iTRAQ labels (AB Sciex Pte Ltd.) were suspended in 100% ethanol, added to the peptide mixtures, and incubated ON at RT. After quenching the reactions with sterile MilliQ water the mixtures were dried and stored at −20°C until use.

### Liquid chromatography and mass spectrometry

iTRAQ-labeled tryptic peptide samples (100 µg) were fractionated by high-pH, C_18_-reversed phase liquid chromatography on a micro-flow Agilent 1100/1200 series system (Agilent Technologies), using a Waters XBridge C_18_ guard column (10 mm long, 2.1 mm inner diameter, 3.5 µm particles) and a Waters XBridge C_18_ analytical column (10 cm long, 2.1 mm inner diameter, 3.5 µm particles). Mixed peptides were dried and suspended in LC buffer A (20 mM ammonium formate, pH 10), then resolved by a gradient of LC buffer A and buffer B (20 mM ammonium formate and 90% acetonitrile, pH 10). The gradient started at 3% B from 0–10 min, 8–11% B from 10–17 min; 11–60% B from 17–75 min; 95% B from 75–80 min; and 3% B from 80–170 min at a constant flow rate of 150 µl/min. Fractions were collected across the peptides elution profile (10–75 min), dried and resuspended in 40 µl of nano LC buffer A (2% acetonitrile, 0.1% formic acid).

Each fraction was separately analysed using a nano-flow Easy nLC II (Thermo Fisher Scientific) connected in-line to an LTQ Orbitrap Velos mass spectrometer (Thermo Fisher Scientific) with a nano-electrospray ion source (Thermo Fisher Scientific). The peptide fractions (6 µl) were loaded onto a C_18_-reversed phase trap column (2 cm long, 100 µm inner diameter, 5 µm particles) with 100% buffer A (2% acetonitrile, 0.1% formic acid) at 4 µl/min for a total volume of 30 µl, and then separated on a C_18_-reversed phase column (15 cm long, 75 µm inner diameter, 3 µm particles). Both columns were packed in-house with ReproSil-Pur C_18_-AQ resin (Dr. Maisch). Peptides were eluted using a linear gradient of 2–20% buffer B (98% acetonitrile, 0.1% formic acid) over 120 min at a constant flow rate of 250 nl/min. The total LC/MS/MS run-time was 160 minutes, including the loading, linear gradient, column wash at 95% buffer B, and the equilibration.

Data were acquired using a data-dependent method, dynamically choosing the top 10 abundant precursor ions from each survey scan for isolation in the LTQ and fragmentation by HCD at 45% normalized collision energy. The survey scans were acquired in the Orbitrap over *m/z* 300–1700 with a target resolution of 60,000 at *m/z* 400, and the subsequent fragment ion scans were acquired in the Orbitrap over a dynamic *m/z* range with a target resolution of 7500 at *m/z* 400. The lower threshold for selecting a precursor ion for fragmentation was 1000 counts. Dynamic exclusion was enabled using a list size of 500 features, a *m/z* tolerance of 15 ppm, a repeat count of 1, a repeat duration of 30 s, and an exclusion duration of 15 s, with early expiration disabled.

### Data processing

All spectra were processed using Mascot Distiller v2.4.1 (Matrix Science), and database searching was done with Mascot v2.4 (Matrix Science). Searches were performed against an in-house built, non-redundant database consisting of NCBI's Genome database of bacteria [ftp://ftp.ncbi.nlm.nih.gov/genomes/Bacteria/] and prophage sequences generated in-house. The decoy database option was selected and the following parameters were used: carbamidomethylation (C) and iTRAQ (K and N-terminus) as fixed modifications, oxidations (M) as a variable modification, fragment ion mass tolerance of 0.5 Da, parent ion tolerance of 10 ppm, and trypsin enzyme with up to 1 missed cleavage. Mascot search results were imported into Scaffold Q+ v4.0 (Proteome Software) and filtered using 1.0% Protein FDR; 2 peptides; 0.1% Peptide FDR.

## Results

### CJIE4 localization in the chromosome

*C. jejuni* prophages homologous to CJIE4 were detected in several isolates in previous work [8]. Full prophage genomes, lacking only part of the genes on either end of the prophage and the adjacent chromosomal insertion sites, were obtained from *C. jejuni* isolates 00-2425, 00-0949, 00-1597, 00-6200, and 01-1512 by sequencing short (2.5 to 3 kb) regions using PCR primers developed using the DNA sequence of CJIE4 from strain RM1221. These sequences were assembled, annotated, compared, and the resulting DNA and protein sequence data used to find homologous sequences from genomes deposited in sequence databases. Very recently completed draft whole genome sequences of the five isolates were used to unambiguously determine the locations of the prophages in the respective genomes and confirm earlier sequence data (unpublished data).

For isolates 00-0949, 00-1597, 00-2425, and 00-6200, the CJIE4 prophage was located between tRNA-Met and tRNA-Phe as with all of the other CJIE4 prophages (isolate 00-2425, accession number CP006729; for the other isolates, unpublished data). However, in isolate 01-1512 CJIE4 was inserted between Cj0339c (CJE0384) and Cj0341c (CJE0386). To ensure that this was not an artifact caused by mis-assembly of the draft genome, PCR primers were developed for both the upstream and downstream regions of CJIE4, with one primer located within the prophage and the other within the genomic DNA region (see Materials and Methods). Amplicons were obtained for both primer sets from *C. jejuni* 01-1512 but not 00-0949 (data not shown), indicating that the genomic location of CJIE4 was indeed between Cj0339 and Cj0341 and that the prophage had translocated at some time from one location to another. tRNA-Met was found in 00-0949, 00-1597, 00-2425, and 00-6200 upstream flanking sequences as it was previously in strain RM1221 [1]. Only the last 17 nucleotides of tRNA-Met 01-1512 were detected upstream of the CJE1418 gene, indicating that the translocation of the prophage or some subsequent event disrupted this tRNA locus.

### CJIE4 exhibits the heterogeneity common to bacteriophages

Searches of the NCBI Whole-genome shotgun contigs (wgs) database with the entire nucleotide sequence available for CJIE4 from 00-0949 and 00-2425 revealed two homologous prophages with extensive regions of identity to the five prophages sequenced in the current study. CJIE4 prophages were detected in additional strains by performing BLAST searches in GenBank using a number of protein sequences from the five strains sequenced in this work. In total, 12 of these additional CJIE4 sequences were used for further comparisons.

**Figure 1:**
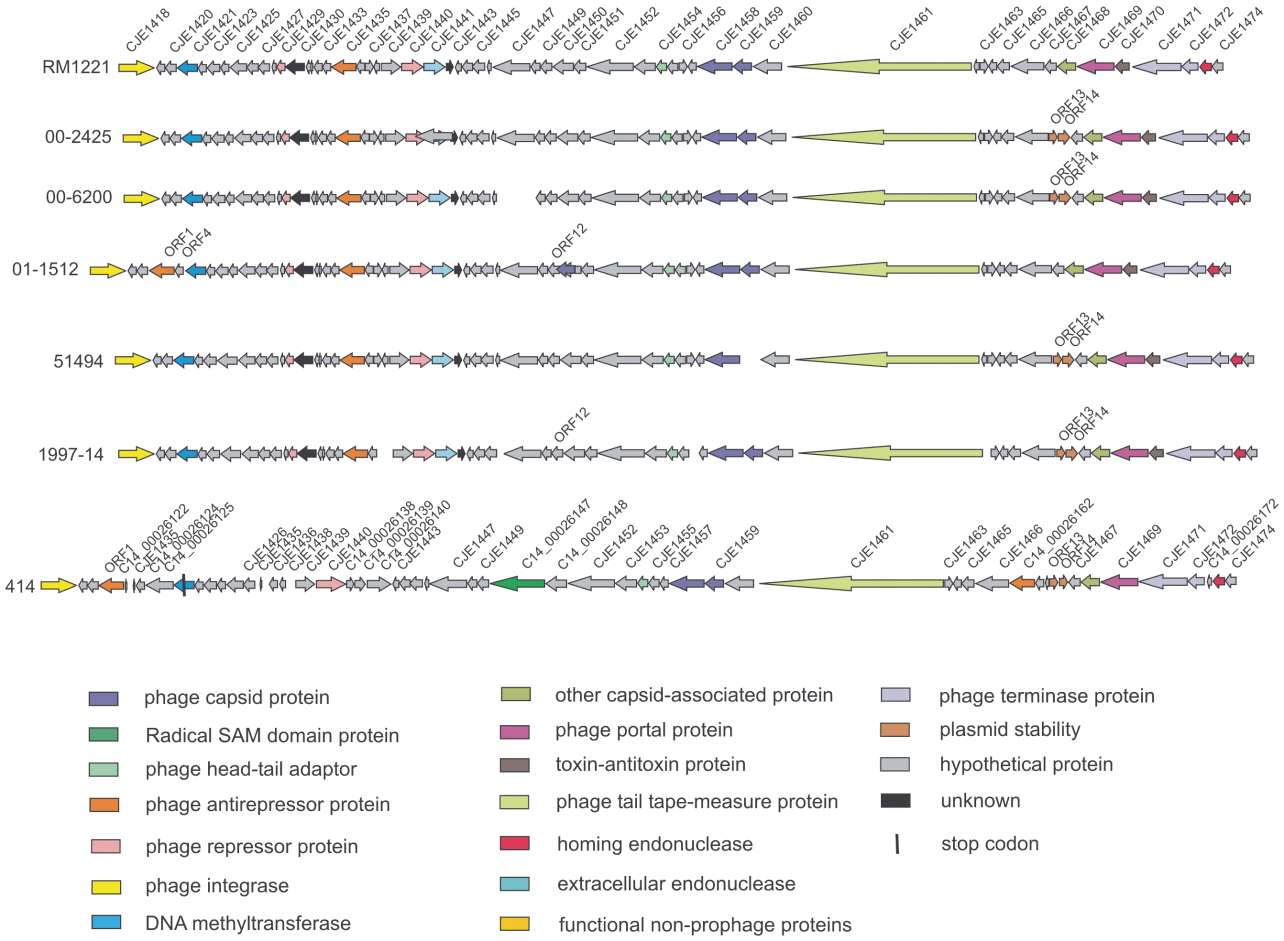
Schematic diagram demonstrating differences in Group 1 CJIE4 prophage DNA sequences. Strains carried the large indel containing genes encoding CJE1439-Cj1442, plus strain 414. The diagram includes prophages investigated in this work (see Materials and Methods) plus those obtained from NCBI databases. Sizes of genes are approximate, and any non-coding sequence between genes has not been shown to conserve space.

**Figure 2:**
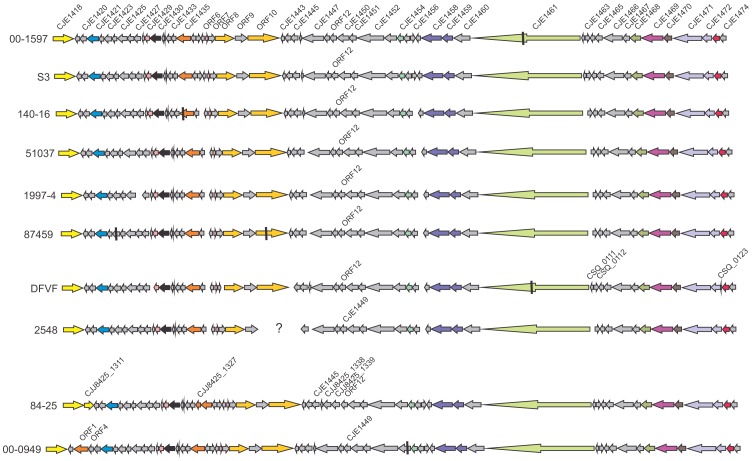
Schematic diagram demonstrating differences in Group 2 CJIE4 prophage DNA sequences. Strains carried the alternate large indel containing ORFs 6-10. The diagram includes prophages investigated in this work (see Materials and Methods) plus those obtained from NCBI databases. The question mark in the 2548 indicates that the fact that no sequence was found for this region may indicate either that there was a problem with the search parameters used to detect contigs encoding proteins homologous to those in other CJIE4 variants or that there was a horizontal gene transfer replacing the region with a different gene repertoire. The colors used to identify putative protein functions were the same as in Fig. 1. Sizes of genes are approximate, and any non-coding sequence between genes has not been shown to conserve space.

The five prophages newly sequenced for this study showed extensive regions of identity among themselves as well as with CJIE4 from strains RM1221 and S3 (Figures 1 and 2), which appeared to be prototypical phages encompassing much of the variability encoded within this temperate bacteriophage family. There were two major groups of prophage defined by an indel encoding homologs of four RM1221 genes between CJE1438 and 1443 in one group of isolates (Group 1 sequences; Table 1, Figure 1) and five genes designated, for the purposes of this discussion, ORF6 - 10 in the second group of organisms (Group 2 sequences; Table 1, Figure 2). This latter indel had 100% nucleotide identity over 4097/4097 nt with a sequence in *C. jejuni* S3, which was therefore considered the prototype prophage for this group. Genes within each of the two indels in both Group 1 and Group 2 prophage sequences were transcribed in the direction opposite to the remaining prophage genes. A *C. coli* strain, 2548, exhibited a 2092 nt region of complete identity and a second region with partial (87%; 410/473 nt) identity within the 4097 nt indel (Figure 2). There were lower levels of identity with the upstream insertion site for CJIE1 (80% identity over 496/619 nt) containing a partial sequence of *panB*, as well as with the KAP-family protein also present at the left end of CJIE1 [7].

**Table 1:** Comparison of sequence annotation for five CJIE4 prophages.

RM1221		Presence (+) or absence (-) in isolate or prophage				
homolog	Gene/protein description	00-2425	00-6200	01-1512	00-1597	00-0949
CJE1418	*intA*, phage integrase, site-specific recombinase	+	+	+	+	+
CJE1419	DNA binding protein, transcriptional regulator (excisionase)	+	+	+	+	+
CJE1420	hypothetical protein	+	+	+	+	+
ORF1	RHA family protein; antirepressor?	-	-	+	-	+
ORF4	hypothetical protein	-	-	+	-	+
CJE1421	*dpnA*, site-specific DNA methyl-transferase	+	+	+	+	+
CJE1422	Emm-like protein	+	+	+	+	+
CJE1423	hypothetical protein	+	+	+	+	+
CJE1424	hypothetical protein	+	+	+	+	+
CJE1425	hypothetical protein	+	+	+	+	+
CJE1426	hypothetical protein	+	+	+	+	+
CJE1427	hypothetical protein	+	+	+	+	+
CJE1428	hypothetical protein	+	+	+	+	+
CJE1429	hypothetical protein, transcriptional regulator domain	+	+	+	+	+
CJE1430	RloG protein	+	+	+	+	+
CJE1431	hypothetical protein	+	+	+	+	+
CJE1432	hypothetical protein	+	+	+	+	+
CJE1433	hypothetical protein	+	+	+	+	+
CJE1434	YopX protein	+	+	+	+	+
CJE1435	DNA binding protein Roi, antirepressor domain	+	+	+	+	+
CJE1436	hypothetical protein	+	+	+	+	+
CJE1437	hypothetical protein, 77 aa, opposite strand to CJE1438	+	+	+	+	+
CJE1438	hypothetical protein, 67 aa, opposite strand to CJE1437	+	+	+	+	+
ORF6	bacteriophage CI repressor protein	-	-	-	+	+
ORF7	hypothetical protein	-	-	-	+	+
ORF8	Exonuclease, DNA polymerase III epsilon subunit	-	-	-	+	+
ORF9	hypothetical protein	-	-	-	+	+
ORF10	KAP family P-loop protein	-	-	-	+	+
CJE1439	hypothetical protein	+	+	+	-	-
CJE1440	signal peptidase I, phage repressor protein	+	+	+	-	-
CJE1441	DNA/RNA non-specific endonuclease, *nucA*	+	+	+	-	-
CJE1442	hypothetical protein, PLDc_N superfamily domain	+	+	+	-	-
CJE1443	hypothetical protein, homolog of CJE0599	+	+	+	+	+
CJE1444	hypothetical protein, homolog of CJE0598	+	+	+	+	+
CJE1445	hypothetical protein, homolog of CJE0597	+	+	+	+	+
CJE1446	hypothetical protein, homolog of CJE0596	+	+	+	+	+
CJE1447	hypothetical protein, homolog of CJE0595	+	+	+	+	+
CJE1448	hypothetical protein, homolog of CJE0229 and CJE0594	+	+	+	+	+
CJE1449	hypothetical protein, homolog of CJE0593	+	+	-	-	+
ORF12	hypothetical protein	-	-	+	+	-
CJE1450	hypothetical protein, homolog of CJE0230 and CJE0592	+	+	+	+	+
CJE1451	hypothetical protein, homolog of CJE0591	+	+	+	+	+
CJE1452	hypothetical protein, homolog of CJE0590	+	+	+	+	+
CJE1453	hypothetical protein	+	+	+	+	+
CJE1454	phage head-tail joining protein	+	+	+	+	+
CJE1455	hypothetical protein	+	+	+	+	+
CJE1456	hypothetical protein, 90 aa, opposite strand to CJE1457	+	+	+	+	+
CJE1457	hypothetical protein, 83 aa, opposite strand to CJE1456	+	+	+	+	+
CJE1458	HK 97 family major capsid protein	+	+	+	+	+
CJE1459	HK97 phage prohead protease	+	+	+	+	+
CJE1460	hypothetical protein	+	+	+	+	+
CJE1461	tail tape-measure protein	+	+	+	+	+
CJE1462	hypothetical protein	+	+	+	+	+
CJE1463	hypothetical protein	+	+	+	+	+
CJE1464	hypothetical protein	+	+	+	+	+
CJE1465	DNA repair protein rad2	+	+	+	+	+
CJE1466	hypothetical protein	+	+	+	+	+
ORF13	hypothetical protein	+	+	-	-	-
ORF14	addiction module toxin, RelE/StbE family	+	+	-	-	-
CJE1467	hypothetical protein	+	+	+	+	+
CJE1468	HK 97 phage protein	+	+	+	+	+
CJE1469	HK 97 portal protein	+	+	+	+	+
CJE1470	toxin-antitoxin protein, toxin MazF	+	+	+	+	+
CJE1471	phage terminase, large subunit	+	+	+	+	+
CJE1472	phage terminase, small subunit	+	+	+	+	+
CJE1473	phage HNH endonuclease	+	+	+	+	+
CJE1474	hypothetical protein	+	+	+	+	+

The CJIE4 homolog from strain 414 (Figure 1) appeared to represent a third member of the CJIE4 prophage family due to the much greater number of gene substitutions compared with other phages. Though it carried CJE1439 and the CJE1440 locus characteristic of the RM1221 group of strains it did not have any of the remaining genes from this indel, instead bearing three additional, unrelated genes transcribed in the same direction as CJE1439 and CJIE1440 (Figure 1).

While the two indels described above appeared to define major CJIE4-family prophage groups, there was additional extensive variability in the prophages. Three isolates (*C. jejuni* 00-0949, 01-1512, 414) carried insertions between CJE1420 and 1421 homologues (Figures 1 and 2). The insertion in strain 414 was larger than that of the other two isolates and had four ORFs in addition to the putative antirepressor protein all three prophages had in common. An apparent partial duplication of CJE1418 was found adjacent to the full-length gene in the CJIE4 prophage from strain 84-25. Genes encoding CJE1437, CJE1446, CJE1456, and CJE1463 were not annotated in several phage genomes, and CJE1438 was additionally not found in the 1997-14 CJIE4 prophage genome. A novel gene, designated ORF12 for discussion purposes, replaced CJE1449 in many prophages. The predicted protein sequences of ORF12 and CJE1449 had no significant identity with each other.

Two genes (ORFs 13 and 14) encoding proteins associated with the plasmid stability system were located between CJE1467 and CJE1468 in CJIE4 prophages from isolates 00-2425, 00-6200, 5194, 1997-14, and 414 (Figure 1); none were found in prophages from Group 2 prophages (Figure 2). Premature stop codons were detected in several genes (see Figure 2) and some proteins were annotated as having a different size than the consensus of the group (data not shown). Strain 414, in addition to the differences discussed above, has two genes replacing CJE1450 and 1451 and a four gene insertion between CJE1466 and CJE1467. The prophage from this strain, perhaps to compensate, did not carry the gene encoding the CJE1468 capsid-associated protein (Figure 1).

Two potential prophage repressors were identified on the basis of protein sequence homology with other proteins identified as repressors in NCBI databases. Genes for ORF6 and CJE1440 were present in the two separate indels at the same location within the CJIE4 prophage genome. A gene encoding CJE1440 was also present in strain 414 (Figure 1) even though the indel was different in this strain, including CJE1439 and three additional genes not found in the other CJIE4 DNA sequences included in this study. All CJE1440 and all ORF 6 protein sequences were identical. Neither of the two putative repressor proteins exhibited detectable protein or DNA sequence identity with each other and these two proteins appeared very different to each other based on physicochemical properties inferred from their DNA sequences (Table 2). Both appeared to carry domains in common with the lambda CI repressor but not the lambda Cro repressor.

**Table 2:** Comparison of putative phage repressor proteins associated with CJIE4 with lambda repressors.

Protein	Length	M.W.	Charge	Predicted	Functional Domains
	(a.a.)	(Da)		pI	
lambda Cro	67	7,363.46	+5.07	9.78	Cro superfamily
CJE1440	220	25,472.02	+1.30	7.74	Peptidase_S24_S26 superfamily
ORF6	127	14,777.86	−0.08	6.76	Phage_CI_repr
lambda CI	237	26,211.92	−5.98	4.99	Peptidase_S24_S26 superfamily; HTH_XRE superfamily

### Expression of proteins from both of the two alternate indels was detected in iTRAQ experiments

Expression of CJIE4 prophage proteins was assessed in iTRAQ experiments with isolates grown on Mueller-Hinton agar. The intent was to determine whether any of the proteins were expressed when the prophage was integrated into the chromosome, which may therefore contribute in some way to the biology of the host bacterium in the absence of prophage induction. Proteins were detected previously in iTRAQ experiments for 00-2425 and the results have been communicated (Clark et al., submitted for publication). A second four-plex iTRAQ experiment was conducted in triplicate (three biological replicates) to assess protein expression in the remaining four *C. jejuni* isolates: 00-0949, 00-1597, 00-6200, and 01-1512. The detection of CJIE4-associated proteins from all five isolates, stripped of relative quantitation data and scored only on the basis of whether the proteins were identified or not, is summarized in Table 3.

**Table 3:** Detection of proteins associated with the CJIE4 prophage after growth on Mueller-Hinton agar in iTRAQ labelling experiments.

Identified Protein	Accession Number	00-2425	00-6200	00-1512	00-1597	00-0949
site-specific recombinase, phage integrase family CJE1418	gi|384443628	+	+	+	+	+
hypothetical protein CJE1420, putative excisonase	gi|384443630	-	+	+	+	+
emm-like protein CJE1422	gi|384443632	-	+	+	±	+
hypothetical protein CJE1429	gi|57238165, gi|384443638	+	+	+	+	+
RloG protein, putative CJE1430	gi|384443639	+	+	+	+	+
hypothetical protein CJE1432	gi|57238168, gi|384443641	+	+	+	+	+
hypothetical protein, phage repressor protein ORF6	gi|384443648	-	-	-	+	+
hypothetical protein ORF7	gi|384443649	-	-	-	+	+
exonuclease, DNA polymerase III epsilon subunit ORF8	gi|384443650	-	-	-	+	+
hypothetical protein ORF9	gi|384443651	-	-	-	+	+
KAP family P-loop domain protein ORF10	gi|384443652	-	-	-	+	+
hypothetical protein CJE1439	gi|57238175	+	+	+	-	-
signal peptidase I, phage repressor protein CJE1440	gi|57238176	+	+	+	-	-
DNA/RNA non-specific endonuclease CJE1441	gi|57238723	+	+	+	-	-
hypothetical protein CJE1444	gi|384443653	-	-	+	-	+
hypothetical protein CJE1447	gi|384443655	-	+	+	+	++
hypothetical protein CJE1452	gi|57238311, gi|384443660	+	+	+	+	++
major capsid protein, HK97 family CJE1458	gi|384443665	+	+	±	+	++
DNA repair protein rad2, CJE1465	gi|384443671	-	+	+	+	++
hypothetical protein CJE1466	gi|57238325, gi|384443672	+	+	+	+	+
toxin-antitoxin protein MazF, CJE1470	gi|57238328, gi|384443676	+	+	+	+	+

Expression of proteins from *C. jejuni* isolate 00-2425 was assessed in previous work [10]; -, not detected.

The protein products of ORFs 6–10 were all detected in isolates 00-0949 and 00-1597 but not in the other three isolates. Similarly, the protein products of 3 of 4 genes in the alternate indel (CJE1339, CJE1440, and CJE1441) were detected in isolates 00-2425, 00-6200, and 00-1512. Expression of each of the putative CI-like repressors (ORF6 and CJE1440) was detected. These results were consistent with the DNA sequences obtained for these CJIE4 prophages. The expression of 12 other CJIE4 proteins could be detected in the iTRAQ experiment that included isolates 00-0949, 00-1597, 00-6200, and 01-1512, while only 8 were detected in the iTRAQ experiments that included isolate 00-2425. Among the proteins detected were: CJE1429, which contains a domain associated with transcriptional regulators; the prophage integrase and putative excisionase proteins; the major capsid protein; RloG; and emm-like protein. This suggests some level of derepression of protein expression. Three proteins homologous to CJE1444, CJE1447, and CJE1452 from strain RM1221 were extremely similar to proteins encoded by genes present in CJIE2 [1]; it was therefore not possible to determine whether these proteins were expressed from CJIE4, CJIE2, or both inserted elements. The toxin-antitoxin system protein MazF was detected in all five isolates tested, while the RelE protein was not detected in either isolate 00-2425 or isolate 00-6200. Additional hypothetical proteins with unknown function (CJE1432, CJE1466) were also detected.

### Expression of proteins from other *Campylobacter jejuni* integrated elements (CJIEs)

Previous work has dealt extensively with the expression of CJIE1 proteins under various conditions in isolate 00-2425 (manuscript submitted for publication). Data from those experiments have been included in Table 4. Some differences in protein detection were found in different iTRAQ experiments; only data pertaining to proteins also detected in the iTRAQ experiments with isolates 00-0949, 00-1597, 00-6200, and 01-1512 were included in the table. It was apparent from the relative expression levels in iTRAQ four-plex experiments that the CJIE1 prophage, including the unique moron containing ORF11 [7], was present in isolates 00-0949 and 01-1512 (Table 4). In isolate 00-0949 most of the CJIE1 genes, including the gene encoding a homolog of the CJE0256 dns extracellular deoxyribonuclease, were found throughout 7 of the 25 contigs in the draft genome sequence. Similarly, the gene encoding CJE0256 and all CJIE1 genes except those encoding CJE0262-CJE0264 were detected in 8 of 25 contigs comprising the draft genome of isolate 01-1512. The only CJIE2 genes detected in draft genome sequences were those that were also common with CJIE4 (CJE0590 – CJE0598 [1]), and it was clear from the context of the remaining genes in the contig that these genes belonged to CJIE4 and that CJIE2 was not present in any of the isolates. Consistent with the inability to demonstrate the presence of CJIE2, no expression of CJIE2 proteins was detected in any of the isolates used for this study.

**Table 4:** Detection of proteins associated with the CJIE1 prophage after growth on Mueller-Hinton agar in iTRAQ labelling experiments.

Identified Protein	Accession Number	00-2425	00-6200	00-1512	00-1597	00-0949
ORF11 hotspot protein	gi|544061942	+	-	+	-	+
phage repressor protein CJE0215	gi|57237226, gi|157414969, gi|384443061	+	-	+	-	+
hypothetical protein CJE0216	gi|57237227	-	-	+	-	+
hypothetical protein CJE0217	gi|57237283, gi|384443059	-	-	+	-	+
major tail sheath protein CJE0227	gi|315124253	-	-	+	-	+
extracellular deoxyribonuclease CJE0256	gi|57237266; gi|315124233	+	-	+	-	+
hypothetical protein CJE0262	gi|384443020	-	-	+	-	+
transcriptional regulator CJE0272	gi|57237282	+	-	+	-	+
hypothetical protein CJE0273	gi|57237283	-	-	+	-	+

## Discussion

CJIE4 temperate bacteriophages showed considerable variability, and the genes associated with the largest indels were expressed. Expression of proteins associated with other genes differentially carried in CJIE4 was also detected in comparative proteomics experiments using iTRAQ labeling. Differential production of prophage proteins could potentially result in differential virulence, different biological properties of the *Campylobacter* isolates, or both. An examination of the literature suggests that many of the moron proteins associated with the CJIE4 prophage may have had roles that affected the biology of the phage itself in addition to roles in the biology of the host bacterium. Little has been published about these proteins in *C. jejuni*, so that it is necessary to attempt to infer possible effects on *C. jejuni* biology from what is known about these proteins in other bacteria.

Prophage-encoded DNases, such as the CJE1441 protein, have previously been shown to be expressed, functional, and to result in a non-naturally transformable phenotype leading to stable *Campylobacter* lineages [5], [6]. Expression of the CJIE4 variant of this DNase was detected in the three isolates carrying the gene encoding it (Table 3). A second periplasmic DNase encoded by a gene within the CJIE1 prophage (*dns*, CJE0256) was also detected in isolates 00-0949, 00-2425, and 01-1512 (data not shown). This indicates that 00-0949 and 01-1512 carry the CJIE1 prophage in addition to isolate 00-2425, a supposition borne out by the observation that they also express four other CJIE1 proteins whereas isolates 00-1597 and 00-6200 do not. Whole genome sequencing verified these observations (unpublished data). The absence of the gene encoding the CJE1441 DNase from isolates carrying the second CJIE4 large indel may not have detectable biological consequences in isolates with DNases encoded by prophage CJIE1 or integrated element CJIE2, which carries a DNase (CJE0566) similar to CJE1441 [1]. However, in the absence of CJIE1 and CJIE2 elements, isolates carrying CJIE4 with the indel lacking CJE1441 may not have the ability to inhibit natural transformation and consequently may not form stable lineages [5]. It could therefore be important to know the content of prophage-encoded periplasmic endonucleases in order to interpret population studies of *Campylobacter* spp., perhaps even to assess and interpret data associated with outbreaks in human populations.

The genes encoding two distinct CJIE4-encoded putative CI-like prophage repressor proteins were present, and proteins expressed by these genes were detected in each of the five isolates tested, suggesting either redundancy of function or fine control of maintenance of lysogeny in these isolates. In bacteriophage λ the Cro protein is required for entry into the lytic cycle. None of the proteins identified in the five CJIE4 prophages characterized here had a Cro protein domain. The detection of the major capsid protein indicated that some CJIE4 prophages may be partially or wholly derepressed, though it is not clear whether or not they entered the lytic cycle and produced infectious phage particles. A protein with a function similar to Cro may be expressed, or there may be leaky expression of the capsid protein in the absence of prophage induction. Alternatively, CJIE4-family prophages may regulate switching between lysogeny and the lytic cycle differently than lambda phage.

A toxin-antitoxin protein (CJE1470) was among the CJIE4 prophage-encoded proteins detected. It was highly conserved among all strains investigated except strain 414 and contained PemK and MazF domains associated with type II toxin-antitoxin systems. Type II toxin-antitoxin systems have two genes, a toxin and an antitoxin, forming an operon; both genes are constitutively transcribed [19]. A gene encoding the second protein of this system, MazE, was not detected in the CJIE4 prophage. By cleaving specific mRNA sequences at ACA sites through a ribosome-independent mechanism when activated, MazF systems selectively inhibit the synthesis of a subset of ACA-containing proteins. This endoribonuclease has been proposed to regulate bacterial cell growth in response to stress [19], [20], but is also a functional mRNA interferase in yeast and mice [20]. In *E. coli* MazF also cleaves leader sequences from some mRNAs, creating leaderless mRNAs, and also cleaves the anti-Shine-Dalgarno sequence from 16S RNA within 30S ribosomal subunits. The net result is to generate a distinct subpopulation of ribosomes capable of translating leaderless mRNA, coupling protein synthesis to the physiological state of the bacterial cell [21]. Determination of whether this mechanism is also in *C. jejuni* requires further investigation. A second toxin-antitoxin system, composed of genes we have, for convenience, termed ORF13 and ORF14, was present in five of the sequences examined. However, the proteins were not expressed in detectable quantities. The ORF13 protein product had somewhat limited homology to CopG family transcriptional repressors while the ORF14 protein contained RelE and Plasmid_stabil superfamily domains. RelE toxin-antitoxin systems non-selectively inhibit protein synthesis when activated [19]. The functions of *C. jejuni* toxin-antitoxin systems have not been elucidated, though in *E. coli* activation of one or more of these systems in response to stress could lead to a state of dormancy associated with the ability to survive in the presence of antibiotics. Toxin-antitoxin systems have been associated with enhanced prophage stability [22], suggesting they may play a role in maintenance of the phage(s) upon which they are encoded. *E. coli* toxin-antitoxin systems are necessary for cell death required for biofilm formation [23]. Further experiments will be necessary to determine whether the CJIE4-encoded toxin-antitoxin proteins may have similar roles or functions in *C. jejuni*.

KAP family P-loop proteins include many proteins that confer immunity to bacteriophages [24]. In prokaryotes the genes encoding these proteins appear to have been spread by plasmids, though one is present on a filamentous phage in *Vibrio*. They also appear to have been frequently subjected to recent pseudogene formation, which, in turn, may be associated with phages driving resistance to KAP-mediated pathways [23]. The ORF10 KAP family P-loop protein encoded by CJIE4 was homologous to the CJIE1 KAP family P-loop protein encoded by *C. jejuni* 00-2425 CJIE1, exhibiting 39% amino acid identity, 55% conserved amino acids, and 12% gaps over 591 amino acids with an e-value of 2e-105. On the basis of the literature cited, we hypothesize that the presence of related KAP family P-loop NTPases with different primary amino acid sequences on two different prophages suggests these proteins may have evolved to confer superinfection immunity to specific prophages. An alternate possibility is that they may function to make bacteria immune to infection by lytic bacteriophages. The role(s) of these proteins in the biology of both temperate bacteriophage and the host bacteria should provide a fruitful avenue of further research.

The DNA polymerase III epsilon subunit of *E. coli* has proof reading activity essential for high fidelity DNA synthesis mediated by DNA polymerase III [25]. Silencing of *dnaQ* gene expression reduced the growth rate of *E. coli*, indicating that the product, DNA polymerase III epsilon subunit, affected the processivity (rate of DNA synthesis) of DNA polymerase III [26]. Overexpression of the epsilon subunit may facilitate its interaction with DNA polymerases capable of replicating imperfect DNA templates [27]. This subunit is also implicated in the induction of the *E. coli* SOS response to nalidixic acid [28]. *C. jejuni* strain RM1221 has a copy of the DNA polymerase III epsilon subunit in its chromosome (CJE0502), though this 233 amino acid protein was quite dissimilar from the ORF8 characterized in this study and present in other *C. jejuni* isolates. Since many *C. jejuni* are quite syntenic, it is quite possible that the isolates examined in this study also have a chromosomally encoded epsilon subunit and that the prophage encoded protein is a duplicate; whole genome sequencing studies of the isolates used for this work are underway and should provide an unambiguous answer. BLASTp alignments of ORF8 and CJE0502 performed using the tools in NCBI returned an Expect value of 3e-19 with only 38/94 identities and 60/94 positives, with two gaps. Both ORF8 and CJE0502 had even lower identity with the *E. coli* K12 DNA polymerase III epsilon subunit (Accession no. YP_488512.1, gi|388476328). It is not clear that the functions described above for the *E. coli* DNA polymerase III epsilon subunit would be retained by either of the *C. jejuni* counterparts. Given the differences between ORF8 and CJE0502, it is quite possible that the function(s) of these two proteins could be different, and that these differences may have effects on the biology of *C. jejuni*. These topics require further study.

The hypothetical proteins CJE1439, ORF7, ORF9 encoded by genes in the large indel do not yet have well-defined or well-known functions, but their expression was detected. How these genes or proteins may affect either the CJIE4 prophage or the bacterial host is unknown.

While most of the protein annotations were derived from the use of bioinformatics tools, some proteins were annotated on the basis of other characteristic properties widely shared among temperate bacteriophages. The homologs of CJE1461 were annotated as tail tape measure proteins based on their length, high content of 14 alpha-helical coiled-coil regions, and position in the phage genome relative to the gene encoding the major capsid protein [29], [30]. The phage integrase associated with insertion of phage DNA into the bacterial chromosome is usually linked to an excisionase associated with recovery of the prophage DNA from the bacterial chromosome; the CJE1419 homolog has therefore been annotated as an excisionase.

CJIE2 and CJIE4 were previously shown to carry a module of 10 genes (encoding proteins CJE1443 to CJE1552) that were nearly identical along at least part of their length at the DNA sequence level, though differences in the start site for protein translation did occasionally lead to predicted proteins of different sizes. At least part of this 10 gene module was conserved in all CJIE4 prophages tested to that date [2]. However, several of the loci in this module were variably present, including those encoding CJE1443 and CJE1446-CJE1449.

Comparison of whole genome sequences determined that *C. jejuni* strain 414 was somewhat phylogenetically divergent from most other *C. jejuni* isolates [11]. The CJIE4 prophage from this strain was somewhat different than other CJIE4 prophages as well [31]. It was, for instance, the only CJIE4 prophage that lacked the gene encoding MazF. An examination of the distribution of abnormal tetranucleotide repeats in *Campylobacter* phage genes has suggested that some prophages may have transferred into their hosts a long time ago and subsequently lost the ability to be transferred, while others may still have the capacity for transfer to different hosts 31]. Phage genes within strain 414 had 0% abnormal tetranucleotide repeats [31], strongly suggesting that CJIE4 had undergone an ancient integration event and subsequently became adapted to its host. Whether this is true for other CJIE4 prophages as well will require further investigation.

In summary, the DNA sequence and protein expression data together suggest that moron proteins encoded by genes within the CJIE4 prophage could potentially alter the homeostasis of infected bacteria. This is consistent with previously published data indicating that at least some *C. jejuni* are characterized by high genetic microdiversity resulting from recombination differences in prophage content [32]. It further supports the hypothesis that *C. jejuni* are “generalists” that can exhibit phenotypic diversity in, for instance, their interaction with eukaryotic cells regardless of isolation source and in the absence of large differences in gene content [32]. The differential carriage and expression of moron genes within prophages may be a key component in this diversity, though the mechanisms by which these proteins act requires further elucidation.
